# Fermented Glutinous Rice Extract Mitigates DSS-Induced Ulcerative Colitis by Alleviating Intestinal Barrier Function and Improving Gut Microbiota and Inflammation

**DOI:** 10.3390/antiox12020336

**Published:** 2023-01-31

**Authors:** Kwang-Youn Kim, Jae Dong Son, Su-Jin Hwang, Jong Kwang Lee, Jae Young Park, Kwang Il Park, Tae Woo Oh

**Affiliations:** 1 Korean Medicine (KM)-Application Center, Korea Institute of Oriental Medicine (KIOM), Daegu 41062, Republic of Korea; 2 Department of Veterinary Physiology, College of Veterinary Medicine, Gyeongsang National University, Jinju 52828, Republic of Korea; 3 JKnutra Co., Ltd., B102, 557, Dongtangiheung-ro, Hwaseong-si 18469, Republic of Korea; 4 NewGen Bio Co., Ltd., 991 Worasan-ro, Munsan-eup, Jinju 52839, Republic of Korea; 5 Department of Korean Convergence Medical Science, University of Science & Technology (UST), 1672 Yuseongdae-ro, Yuseong-gu, Daejeon 34054, Republic of Korea

**Keywords:** fermented glutinous rice, inflammation, tight junction, ulcerative colitis, oxidative stress

## Abstract

Ulcerative colitis (UC) is an inflammatory bowel disease caused by various factors, including intestinal inflammation and barrier dysfunction. Herein, we determined the effects of fermented glutinous rice (FGR) on the expression of tight junction proteins and levels of inflammation and apoptosis in the dextran sodium sulfate (DSS)-induced acute colitis model. FGR was orally administered once per day to C57BL/6J mice with colitis induced by 5% DSS in drinking water. FGR administration recovered DSS-induced body weight loss and irregularly short colon lengths. FGR inhibited the DSS-induced decrease in FITC-dextran (FD)-4 permeability and myeloperoxidase activity. Moreover, FGR treatment repaired the reduction of zonula occluden-1 (ZO-1) and occludin expression and the increase in claudin-2 expression in colonic tissue relative to that following DSS administration. FGR treatment significantly recovered expression of cytokines, such as tumor necrosis factor (TNF)-α, interleukin (IL)-6, and IL-1β, in serum or respective mRNA expression in colonic tissue relative to that following DSS administration. FGR regulated levels of oxidative stress-related factors, such as malondialdehyde and glutathione, and the activity of catalase and superoxide dismutase in the colon tissue of the DSS-induced acute colitis mice model. Furthermore, FGR treatment inhibited apoptosis by reducing the activity of caspase-3 and the ratio of Bcl-2 associated X (Bax)/B-cell lymphoma 2 (Bcl-2). Collectively, FGR treatment protected the intestinal barrier from dysfunction and inhibited inflammation and apoptosis in DSS-induced colitis. Therefore, FGR may decrease the inflammatory response and be a candidate for treating and prevention inflammatory bowel disease by protecting the intestinal integrity.

## 1. Introduction

Inflammatory bowel disease (IBD) is a chronic idiopathic inflammatory disease that affects the gastrointestinal tract and consists of two clinical types: ulcerative colitis (UC) and Crohn’s disease [1,2]. Patients with IBD exhibit an increased clinical degree and duration of UC that appears to be closely associated with colorectal cancer development and are reported to have a 1.5–2.4-fold higher colorectal cancer incidence than that of the normal population [3].

UC frequently occurs in European countries and other Western countries, such as United States and Canada, but recently the incidence has been increasing in Asian countries, including South Korea [4]. Symptoms of IBD include abdominal pain, diarrhea, and/or fever, although the cause of these diseases has not been clarified [5]. Substantial evidence shows that the cause of IBD is associated with environmental interactions and genetic and lifestyle factors [6]. The goal of treating UC is to ameliorate mucosal inflammation and induce remission to improve the patient’s quality of life. Steroids, immunomodulators, and 5-aminosalicylic acid (5-ASA) preparations are used today as standard drug treatments, and 20–40% of patients fail drug treatment or undergo a colectomy as a side effect [7].

Several reports have demonstrated that the typical characteristic of IBD is epithelial barrier disruption [8,9]. Recent studies focused on protective epithelial tight junction integrity and decreasing the inflammatory response for IBD treatment [10]. Tight junction integrity plays a critical role in gut permeability for the maintenance of intestinal integrity, metabolism, and immune response [11,12]. The tight junction is consisted of robust junctional complexes such as adhesive transmembrane protein (e.g., claudins and occludin) and intracellular adapter proteins (e.g., zonula occludens (ZO)-1, ZO-2, and ZO-3), and actin cytoskeleton, that establish a physical barrier to protect the luminal microbiota [13].

Glutinous rice has long been used as an antiulcer food in the private sector. Glutinous rice is mentioned as a food for protection of the stomach in Donguibogam, a traditional Korean medical book, and Bonchogangmok, a Chinese herbal medicine book. A recent study showed that glutinous rice extract protects gastric mucosal damage in an ethanol-induced white rat model (The 24th Annual Meeting of the Korean Society for Molecular and Cellular biology T-55). In this study, the anti-inflammatory efficacy of fermented glutinous rice was investigated in a DSS-induced inflammatory bowel disease model. FGR treatment was shown to protect mice against damage induced by DSS administration and restored expression levels of essential tight junction proteins. Furthermore, microbiome analysis indicated that FGR stimulated an increase in the abundance of bacteria beneficial to gut health. This study indicates that FGR treatment has potential for treating UC in humans.

## 2. Materials and Methods

### 2.1. Chemicals and Reagents

Dextran sodium sulfate (DSS) was purchased from MP Biomedicals (Santa Ana, CA, USA) and 5-aminosalicylic acid (5-ASA), hematoxylin, and eosin (H&E) solutions were obtained from Sigma Aldrich (St Louis, MO, USA). ELISA kits for myeloperoxidase (MPO) activity and for mouse tumor necrosis factor-alpha (TNF-α), interleukin-6 (IL-6), and IL-1β were purchased from Thermo Fisher Scientific (Waltham, MA, USA) and eBioscience (San Diego, CA, USA), respectively. Superoxide Dismutase Activity kit, Malondialdehyde (MDA) assay kit, Catalase Activity Kit, and Glutathione Assay Kit were purchased from Thermo Fisher Scientific (Waltham, MA, USA) and Abcam (Cambridge, UK). RIPA lysis buffer and phosphatase were obtained from Millipore (Darmstadt, Germany) and protease inhibitor cocktails were obtained from Roche (Basel, Swiss). BCA protein quantification kit was purchased from Thermo Fisher Scientific and anti-ZO-1, anti-occludin, and anti-F4/80 antibodies were purchased from Santa Cruz Biotechnology (Dallas, TX, USA).

### 2.2. FGR Preparation

Glutinous rice was purchased domestically (Buma Foods Co., Ltd., Yangsan-si, Gyeongsangnam-do, Republic of Korea) and the manufacturing method is as follows. That is, glutinous rice was added and soaked in a three-fold volume of water for 1 h; the mixture was gelatinized at 121 °C for 1 h. Thereafter, saccharification enzyme (Termamyl, Novozymes Korea LTD., Seoul, Republic of Korea) and yeast (Saccharomyces) were added to the gelatinized glutinous rice and saccharification was performed simultaneously at 55 °C for 2 h to obtain saccharified glutinous rice. Then, yeast (Weissella cibaria) was inoculated into the saccharified solution of glutinous rice for primary fermentation, and lactic acid bacteria were inoculated to obtain a secondary fermented product. The FGR produced through this process was used to evaluate the efficacy on DSS-induced colitis.

### 2.3. Animal Studies

**Figure 1:**
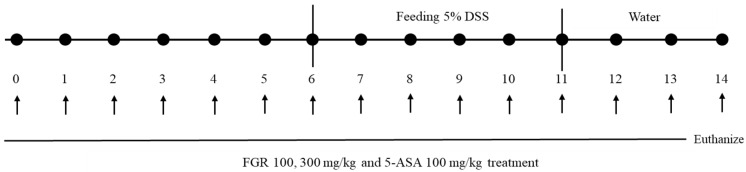
Schematic for DSS-induced colitis animal model. The time course of DSS administration and different treatment in mice. C57BL/6 mice were provided with water or 5% DSS-containing water from day 6 to 11. Before DSS administration, mice were orally administered with different dosages of HDC or 5-ASA.

Male C57/BL6 mice (6 weeks old) were purchased from DooYeol Biotech (Sooul, Korea) and divided into five groups: vehicle-treated control (*n* = 10); 5% DSS (*n* = 10); 5% DSS + FGR 100 mg/kg (*n* = 10); 5% DSS + FGR 300 mg/kg (*n* = 10); and 5% DSS + 5-ASA 100 mg/kg (*n* = 10). In this study, 5-ASA, which was used as a positive control group, is a drug used as an antibiotic to treat ulcerative colitis and Crohn’s disease [14]. During the experimental schedule, mouse body weights were measured daily before the oral administration of FGR (Figure 1). Colonic lengths were measured after the animals were euthanized by inhalation anesthesia with isoflurane. All experimental animal procedures were approved by the Korea Institute of Oriental Medicine Institutional Animal Care and Use Committee (KIOM-21-079) and were conducted in accordance with the guidelines of the National Institutes of Health (NIH publication #83-23, revised in 1985).

### 2.4. Epithelial Paracellular Permeability

A nonabsorbable, FITC-conjugated dextran probe (FD-4) was used to investigate the effects of FGR on paracellular epithelial permeability on the day 8 of the experiment. Mice were gavage fed with 60 mg/100 g body weight FD4 solution. Serum was collected 4 h postgavage feeding, and FD-4 levels were assessed at 490 nm excitation and 520 nm emission wavelengths (VERSAmax microplate reader, Molecular Devices) [15].

### 2.5. Large Intestine Endoscopy and Histological Analysis

To investigate the protective effects of FGR on DSS-induced colitis, high-resolution images of colons were obtained using a miniendoscope with a visible light source (OLYMPUS, Tokyo, Japan; 670-mm length and 2.8-mm diameter) [10]. Following the endoscopy, whole blood and large intestine tissue were obtained from euthanized mice. Colonic tissues were fixed with 4% paraformaldehyde solution, embedded in a paraffin block, and sectioned using a microtome. Histological sections were stained with H&E and PAS or incubated with antibodies to detect the macrophage marker, F4/80, and the tight junction proteins, ZO-1 and occludin [10].

### 2.6. ELISA for Myeloperoxidase (MPO) Activity and Levels of IL-6, TNF-α and IL-1β

Levels of serum IL-6 and TNF-α were assessed using ELISA kits according to the manufacturer’s protocol. In addition, myeloperoxidase activity was determined using MPO activity assay kit according to the manufacturer’s protocol using homogenized colonic tissue.

### 2.7. ELISA for Malondialdehyde (MDA) Content, Catalase Activity, Superoxide Dismutase (SOD) Activity, and Glutathione (GSH) Contents

MDA and GSH contents and catalase and SOD activity were determined using ELISA assay kit according to the manufacturer’s protocol using homogenized colonic tissue.

### 2.8. Terminal Deoxynucleotidyl Transferase dUTP Nick End Labeling (TUNEL) Assay

A TUNEL kit (Beyotime Biotechnology, Beijing, China) was used to assess cell death according to the manufacturer’s instructions, and 4′,6-diamidino-2-phenylindole (DAPI; Sigma, St. Louis, USA) was used for nuclei staining.

### 2.9. Real-Time PCR for Colon Tissue

Colonic tissues were immediately frozen in liquid nitrogen and homogenized with Trizol (Life Technologies, Carlsbad, CA, USA), and total RNA was isolated according to the manufacturer’s instructions. cDNA was synthesized using a RevertAid RT Reverse Transcription Kit (Thermo Fisher Scientific, Waltham, MA, USA). Quantitative real-time PCR (qRT-PCR) was performed using FastSYBR mixture (CWBIO) with specific primers. An internal control was used to normalize to the expression of the *GAPDH* reference.

### 2.10. Western Blot Analysis

Colonic tissues were homogenized using RIPA lysis buffer with phosphatase and protease inhibitor cocktails. The protein content was assessed using a BCA (Bicinchoninic Acid, Waltham, MA, USA) kit and Western blotting was performed as previously described [1,2].

### 2.11. Biochemical Assays

The concentration of malonicdialdehyde (MDA) and glutathione (GSH) and the activity of catalase and superoxide dismutase (SOD) were determined in homogenized colonic tissue using Lipid Peroxidation (MDA) Colorimetric assay kit (Abcam, Cambridge, UK) and catalase, SOD, and GSH assay kits (Cayman, Ann Arbor, MI, USA). The protein concentration was assessed the quantification using a BCA kit.

### 2.12. Intestinal Microbiome Analysis (Metagenomics)

After oral administration for 14 days, mice were euthanized, and intestinal contents were collected at the end point. Intestinal contents were kept on ice during collection and stored at −70 °C until DNA extraction. For intestinal contents, DNA was extracted from each sample using a FastDNA spin kit (MPBio). Amplicon PCR was performed using the 16S rRNA gene and primers (518F, 5′-CCAGCAGCCGCGGTAATACG-3′; and 805R, 5′-GACTACCAGGGTATCTAATCC-3′) for the V4 region of the hypervariable region according to the iSeq library production protocol provided by Illumina. PCR products were purified using Ampure XP, and then indexing PCR was performed using Illumina Nextera XT index kit. After secondary purification, sequencing was performed in a paired-end (151 bp × 2) method using the iSeq 100 platform. Paired-end reads obtained after sequencing were merged and trimmed according to quality criteria. Operational taxonomic units were calculated at 97% sequence-identity cutoff using the VSEARCH algorithm, and taxonomic assignment was performed using RDP classifier (https://rdp.cme.msu.edu/classifier/ accessed on 15 June 2022). α-Diversity and heatmap clustering analysis were performed using MicrobiomeAnalyst (https://www.microbiomeanalyst.ca/MicrobiomeAnalyst/home.xhtml accessed on 10 December 2022).

### 2.13. qRT-PCR Analysis of Beneficial and Harmful Bacteria

For qRT-PCR analysis of beneficial bacteria (*Lactobacillus* and *Bifidobacterium*) and harmful bacteria (*E. coli* and *Clostridium*), 50 ng of DNA extracted from intestinal contents was used as a template, and standard DNA was obtained using 16S rRNA gene-specific primers (Table 1). PCR-amplified products were purified and vector DNA ligated to pGemT-easy vector. The primer sequences and PCR annealing temperatures are shown in the table below. Real-time PCR equipment (MIC, BioMolecular System) was used for real-time amplification of targets, and the experiment was performed using SYBR Green dye.

### 2.14. Statistical Analysis

All graphs were drawn with GraphPad Prism version 5. Experimental values are given as the means ± standard error of the mean. The significant difference was determined using a one-way ANOVA test. A *p*-value < 0.05 were regarded as statistically significant. Post hoc comparison of means was carried out with the Tukey’s test (for one-way ANOVA) or with the Bonferroni’s test (for two-way ANOVA, DSS treatment, FRG treatment and interaction between DSS and FRG administration) for multiple comparisons, when appropriate.

## 3. Results

### 3.1. Effects of FGR on DSS-Induced Body Weight Loss, Colon Length, Permeability, and MPO Activity

**Figure 2:**
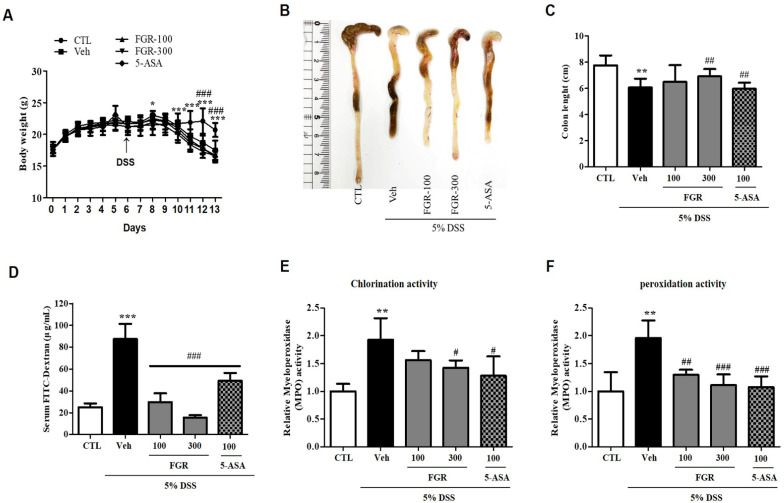
Effects of fermented glutinous rice (FGR) on body weight, colon length, serum FITC-dextran permeability, and myeloperoxidase (MPO) activity in dextran sodium sulfate (DSS)-induced colitis model. (**A**) Body weight, (**B**,**C**) colon length, (**D**) serum FITC-dextran permeability, and (**E**,**F**) myeloperoxidase (MPO) activity. Mice were orally administered with FGR or 5-aminosalicylic acid (5-ASA) (100 mg/kg) before DSS treatment. Body weights were monitored before FGR or 5-ASA treatment. Colonic lengths were measured after excision from euthanized mice. Epithelial paracellular permeability was measured using FITC-conjugated dextran probe (FD-4). MPO activity was measured using an MPO activity assay kit according to the manufacturer’s protocol. Results represent the mean ± standard error of the mean values of each mouse in the same group. *, *p* < 0.05; **, *p* < 0.01; ***, *p* < 0.001 versus the control group and #, *p* < 0.05; ##, *p* < 0.01; ###, *p* < 0.001 versus the DSS only treated group.

We demonstrated the protective effects of FGR on DSS-induced colitis. The body weight of DSS-administered mice was significantly reduced in comparison with that of the control group (Figure 2A). However, mice treated with FGR and 5-ASA showed a clear improvement in body weight loss as compared to that in the DSS-administered mice (Figure 2A). Furthermore, consistent with body weight results, the decrease in colon length was abated by treatment with FGR as well as 5-ASA compared with that in the DSS-administered group (Figure 2B,C). Serum FITC-dextran content in the DSS-administered group (87.27 ± 6.98) markedly increased compared with that in the control group (25 ± 1.74), whereas the serum FITC-dextran content was significantly decreased in the FGR-treated groups (29.70 ± 4.06 or 15.50 ± 1.07) in a dose-dependent manner compared with that in the DSS-administered group (Figure 2D). Additionally, FITC-dextran levels of 5-ASA-treated groups was decreased compared with the DSS-administered group but not when compared with FGR-treated groups. These results indicated that FGR treatment can reduce the increased intestinal permeability present in the UC mice model. In addition, DSS-administered mice had significantly higher MPO activity than mice in the vehicle group for both chlorination and peroxidation activity (Figure 2E,F). However, treatment with FGR and 5-ASA significantly reduced the DSS administration increase in MPO activity (Figure 2E,F).

### 3.2. Effects of FGR on DSS-Induced Histological and Morphological Changes of the Large Intestine

**Figure 3:**
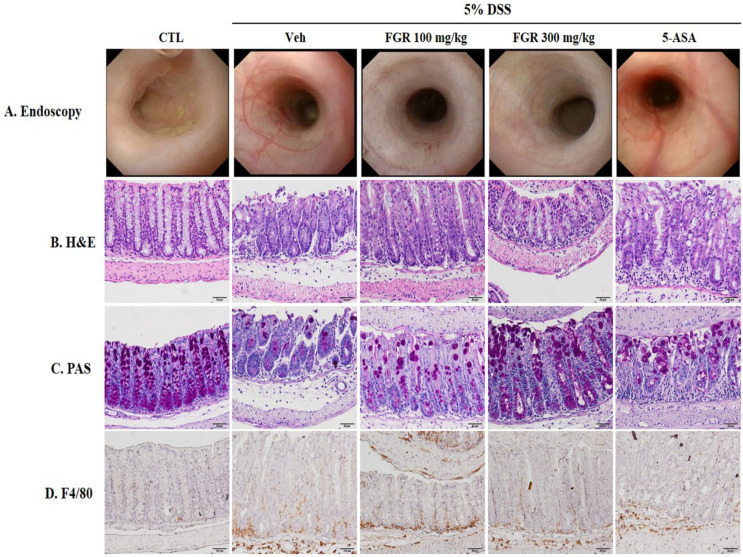
The protective effect of fermented glutinous rice (FGR) in dextran sodium sulfate (DSS)-induced colitis model. (**A**) Endoscopy image, (**B**) hematoxylin and eosin staining (H&E) staining, (**C**) PAS staining, and (**D**) F4/80 staining. On day 8, the DSS-induced mucosal damage was confirmed via miniendoscopy. After the endoscopy, collected intestinal tissues were fixed with 4% paraformaldehyde solution, embedded in a paraffin block, and sectioned using a microtome. Histological sections were stained with H&E, PAS, and F4/80 to determined histological changes. Representative images are shown. Magnification is 200×.

We investigated the effect of FGR treatment via the histological and morphological changes of the large intestine by DSS using endoscopy and H&E and PAS staining. The DSS-administered group exhibited colon tissue damage demonstrated by unhealthy morphological and histological changes, including ulceration, inflammatory cell infiltration, tissue hyperplasia, and crypt atrophy (Figure 3A,B), which recovered with FGR treatment (Figure 3A,B). Furthermore, the reduction in number of goblet cells in the DSS-administered group (Figure 3C) was prevented by FGR treatment. These results indicated that DSS-induced colitis damage could be ameliorated with FGR treatment (Figure 3C). Macrophage infiltration was significantly increased in the DSS-administered group compared with that in the control group, but this was reduced with FGR treatment compared with that in DSS-treated group (Figure 3D). 5-ASA, as a positive control, repaired DSS-induced colon tissue damage and decreased goblet cell numbers consistent with results of FGR-treated groups.

### 3.3. Effects of FGR on Intestinal Barrier Function in Mice with DSS-Induced Colitis

**Figure 4:**
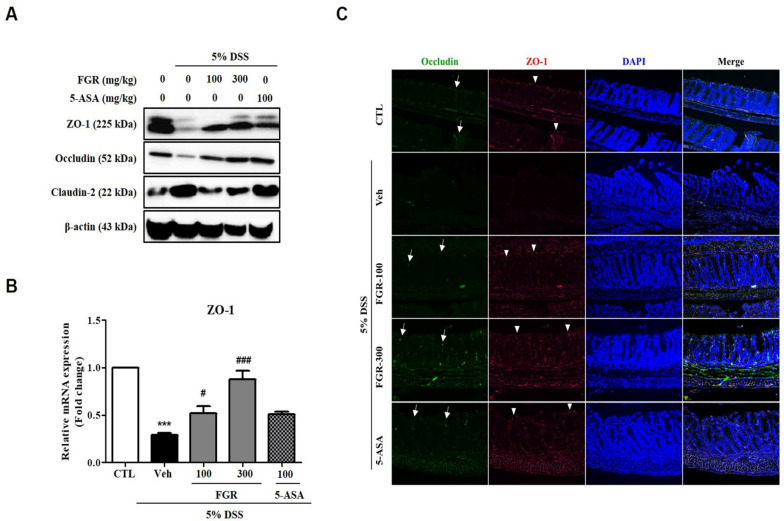
The protective effect of fermented glutinous rice (FGR) on tight junctions in dextran sodium sulfate (DSS)-induced colitis model. (**A**) Representative expression for ZO-1, occluding, and claudin-2 proteins. The expression of ZO-1, occludin, and claudin-2 in colonic tissues was determined by Western blotting; β-actin was used as the protein loading control. (**B**) Relative ZO-1 mRNA expression. (**C**) Representative image of immunofluorescence staining for ZO-1 and occludin expression. Collected intestinal tissues were fixed with 4% paraformaldehyde solution, embedded in a paraffin block, and sectioned using a microtome. Histological sections were stained with anti-ZO-1 (red, arrowheads), occludin (green, arrows) antibody, and DAPI (blue) staining. Data are the mean ± standard error of the mean values of each mouse in the same group. ***, *p* < 0.001 versus the control group and #, *p* < 0.05; ###, *p* < 0.001 versus the DSS only treated group. Magnification was 200×.

Immunofluorescence (IF) staining and Western blot analysis were used to reveal the effects of FGR on the regulation of expression of tight junction proteins, such as ZO-1, occludin, and claudin-2. Expression of ZO-1 and occludin was notably decreased while that of claudin-2 was increased in colon tissue in DSS-administered mice. However, these changes in expression were restored by treatment with FGR (Figure 4A). Moreover, IF staining results were consistent with Western blotting results and showed that the decreased expression of ZO-1 and occludin in DSS-induced colitis was recovered by FGR treatment (Figure 4C). Additionally, the 5-ASA-treated group was increased compared with the DSS-administered group in expression levels of tight junction proteins but less increased compared with FGR-treated groups. Collectively, these results indicate that FGR prevented the DSS-induced acute colitis by regulating of tight junction proteins as a preventive mechanism for colitis.

### 3.4. Effects of FGR on Serum and mRNA Levels of Proinflammatory Cytokines in Mice with DSS-Induced Colitis

**Figure 5:**
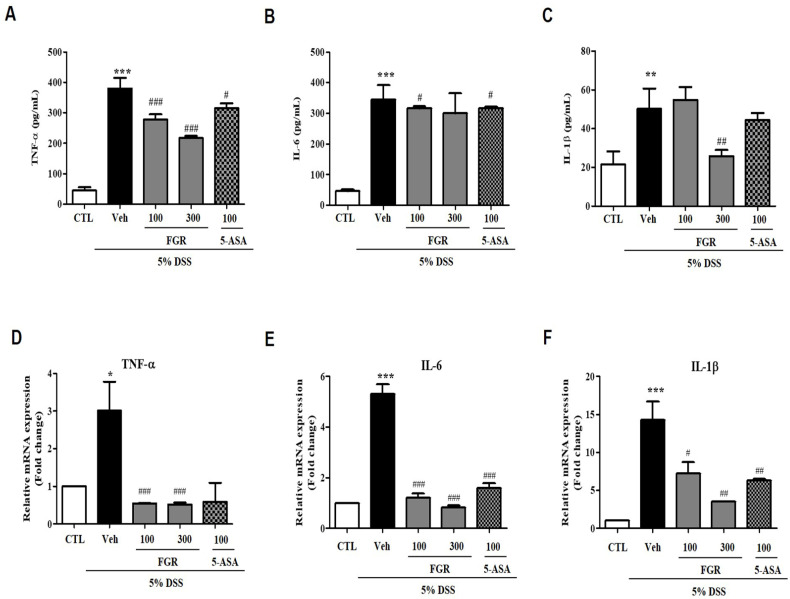
Effects of fermented glutinous rice (FGR) on the serum and mRNA levels of proinflammatory cytokines in dextran sodium sulfate (DSS)-induced colitis model. Serum levels of (**A**) tumor necrosis factor (TNF)-α, (**B**) interleukin (IL)-6, and (**C**) IL-1β. The serum levels of proinflammatory cytokines were detected by ELISA kit for TNF-α, IL-6, and IL-1β. Relative expression of mRNA for (**D**) TNF-α, (**E**) IL-6, and (**F**) IL-1β. Cytokine and mRNA levels results represent the mean ± standard error of the mean values of each mouse in the same group. *, *p* < 0.05; **, *p* < 0.01; ***, *p* < 0.001 versus the control group and #, *p* < 0.05; ##, *p* < 0.01; ###, *p* < 0.001 versus the DSS only treated group.

Several reports showed that proinflammatory cytokines were associated with occurred colitis [16]. Therefore, we measured the serum and mRNA levels of IL-6, TNF-α, and IL-1β (pg/mL), which were significantly higher in the DSS-administered group than those in the vehicle group (Figure 5A–C). However, the serum levels of IL-6, TNF-α, and IL-1b in mice treated with FGR were lower than those in the DSS-treated group (Figure 5A–C). Furthermore, consistent with serum cytokine levels, the expression of mRNA for these cytokines was significantly decreased by FGR treatments compared with that in the DSS-administered group (Figure 5D–F). Furthermore, high doses of FGR treatments were more significantly reduced the serum and mRNA levels of proinflammatory cytokines compared with 5-ASA-treated groups.

### 3.5. Effects of FGR on Oxidative Stress in Mice with DSS-Induced Colitis

**Figure 6:**
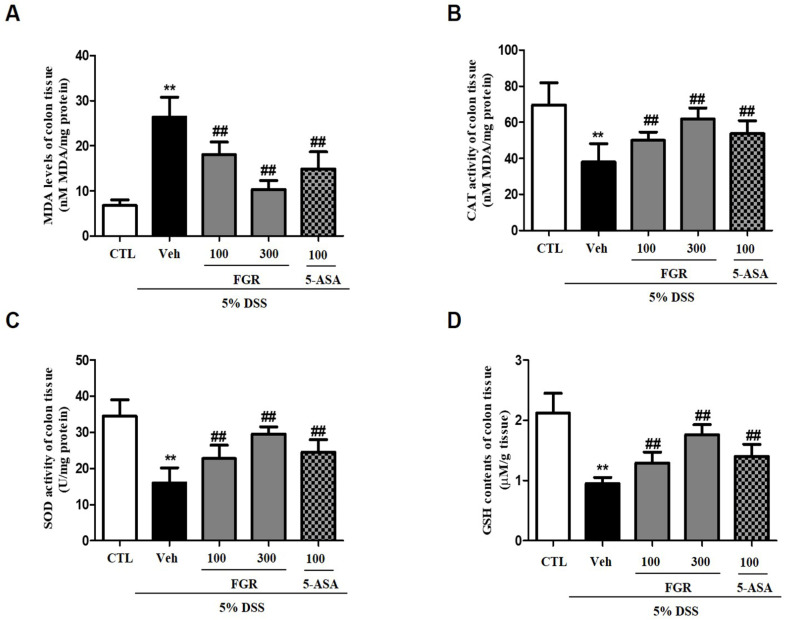
Effect of FGR on oxidative stress-related activities in the colon tissue in dextran sodium sulfate (DSS)-induced colitis model. (**A**) Malondialdehyde (MDA) content, (**B**) catalase activity, (**C**) superoxide dismutase (SOD) activity, and (**D**) glutathione (GSH) contents. Data are presented as mean ± SD, ** *p* < 0.01 compared with control group, and ## *p* < 0.01 compared with the DSS only treated group.

To evaluate the influence of FGR on oxidative stress, such as lipid peroxidation and the antioxidant defense system, in colon tissue, we measured the level of MDA and GSH and the activity of catalase and SOD. Compared with that in the normal group, mice in the colitis model group exhibited a significant increase in the levels of MDA (Figure 6A), whereas the activities of catalase and SOD and levels of GSH were decreased (Figure 6B–D). However, treatment with FGR opposed these changes, restoring the lipid peroxidation concentration and MDA content (Figure 6A) and inhibiting the decrease in GSH content and SOD and CAT activity in colon tissue (Figure 6B–D) in a dose-dependent manner. Furthermore, high doses of FGR treatments more significantly increased the effects of the antioxidant compared with 5-ASA-treated groups.

### 3.6. Effect of FGR on Apoptosis in Mice with DSS-Induced Colitis

**Figure 7:**
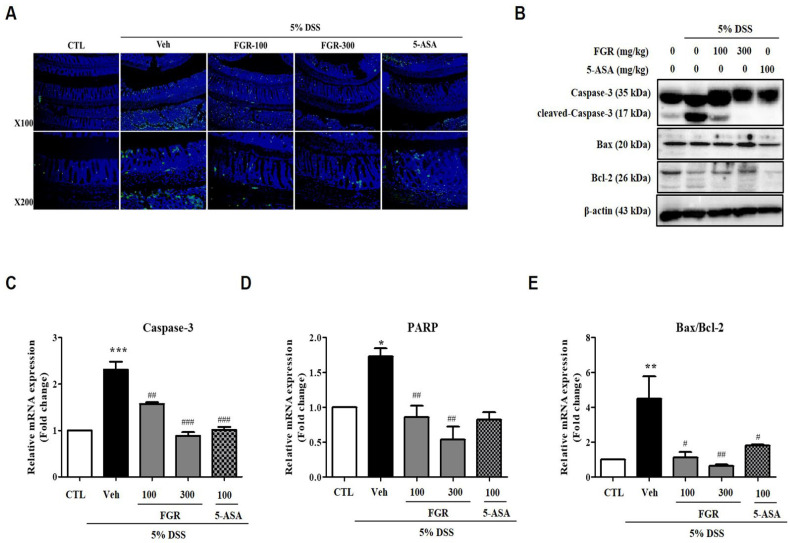
The protective effect of fermented glutinous rice (FGR) on apoptosis induction in dextran sodium sulfate (DSS)-induced colitis model. (**A**) Representative image of TUNEL staining. (**B**) Representative expression of caspase-3, Bax, and Bcl-2 proteins determined by Western blotting; β-actin was used as the protein loading control. (**C**,**D**) Relative expression of mRNA of caspase-3 and PARP. (**E**) The ratio of Bax/Bcl-2 mRNA expression. mRNA levels. Results represent the mean ± standard error of the mean values of each mouse in the same group. *, *p* < 0.05; **, *p* < 0.01; ***, *p* < 0.001 versus the control group and #, *p* < 0.05; ##, *p* < 0.01; ###, *p* < 0.001 versus the DSS only treated group.

TUNEL and Western blot assays were performed using DSS-induced colitis colon tissue to define the protective effects of FGR on DSS-induced apoptosis. As expected, DSS-administered mouse colon tissues clearly exhibited increased induction of apoptosis, whereas FGR effectively suppressed this (Figure 7A). DSS exposure induced an increase in expression of apoptotic proteins compared with that in the vehicle group, whereas FGR significantly suppressed this cell apoptosis (Figure 7B–E). Furthermore, high-dose FGR treatments increased antiapoptotic effects more compared with 5-ASA-treated groups by controlling apoptotic proteins expressions.

### 3.7. Effect of FGR on Intestinal Microbial Composition in Mice with DSS-Induced Colitis

**Figure 8:**
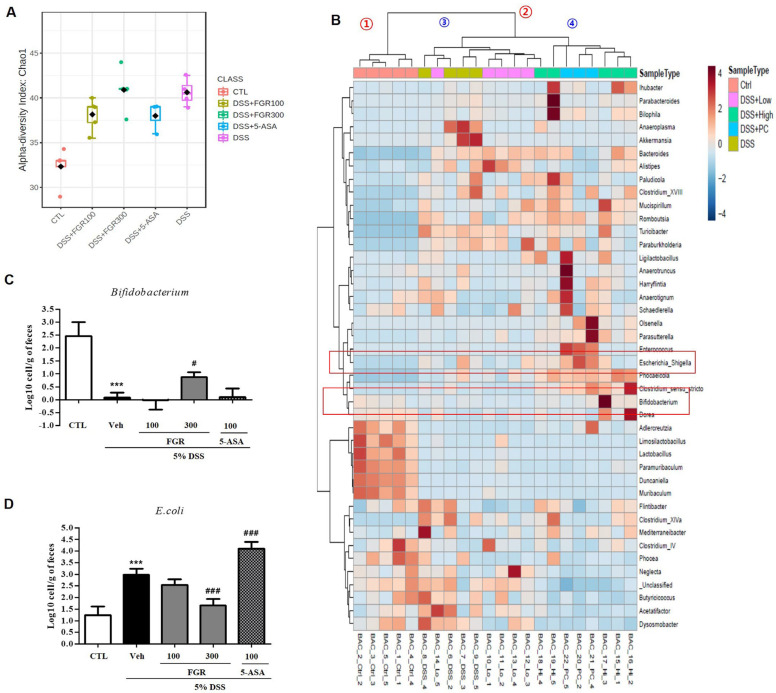
Effect of fermented glutinous rice (FGR) on relative gut bacterial levels in dextran sodium sulfate (DSS)-induced colitis model. (**A**) Genus level α-diversity analysis. (**B**) Heatmap clustering of genus level intestinal flora in DSS-induced colitis model. (**C**) qRT-PCR analysis of beneficial bacteria (*Bifidobacterium*) in DSS-induced colitis model. (**D**) qRT-PCR analysis of harmful bacteria (*E. coli*) in DSS-induced colitis model. mRNA levels are the mean ± standard error of the mean values of each mouse in the same group. ***, *p* < 0.001 versus the control group and #, *p* < 0.05; ###, *p* < 0.001 versus the DSS only treated group.

We performed 16S rRNA assays using DSS-induced colitis intestinal contents to define the protective effects of FGR on metagenomic changes induced by DSS. Analyzing the alpha diversity of the microbial flora at the genus level showed that DSS treatment increased the alpha diversity. DSS caused a significant change in the normal intestinal flora (Figure 8A), which clustered into two main groups: control and DSS treatment conditions (No. 1 and No. 2) based on the relative ratio of the microbial community. Group No. 2 further clustered into two subgroups. The DSS-treated and the low-concentration FGR-treatment groups were classified into one subgroup, whereas the high-concentration FGR treatment group and the positive control group were classified into different subgroups (Nos. 3 and 4). This provides evidence for the concentration-dependent effect of the FGR treatment. The abundance of *Bifidobacterium*, a representative genus of intestinal beneficial bacterial, decreased during DSS treatment but increased in a concentration-dependent manner during FGR treatment. Conversely, the abundance of *Escherichia*, a harmful bacterial genus, significantly increased when DSS was administered but showed a marked decrease after FGR treatment (Figure 8B). We next assessed the presence of the beneficial and harmful bacteria in the DSS model with and without FGR treatment using qRT-PCR. The presence of the beneficial bacteria *Bifidobacterium* mRNA was consistent with metagenomic analysis. *Bifidobacterium* mRNA levels decreased in the DSS-induced group but increased in a concentration-dependent manner during FGR treatment (Figure 8C). The levels of *Escherichia* mRNA increased during DSS treatment but decreased in a concentration-dependent manner during FGR treatment with a statistically significant decrease present in the FGR high-dose group (Figure 8D). However, treatments with 5-ASA were not influenced by the DSS-induced microbial composition change.

## 4. Discussion

IBD is an intractable disease that requires continuous management, and new therapeutic agents are in medical demand. Current therapy can delay the rate of intestinal damage according to drug reactivity and clinical response but is insufficient for treating and preventing chronic diseases. In this study, we revealed the protective effects of FGR against DSS-induced colitis via the regulation of expression of tight junction proteins, such as ZO-1, occludin, and claudin-2, and improvement of intestinal barrier dysfunction. Furthermore, we showed that body weight loss, shortened colon length, crypt damage, and decreased intestinal permeability were restored by treatment with FGR.

Excessive oxidative stress and abnormal production of proinflammatory cytokines are associated with colitis pathogenesis [17,18,19] with the aberrant release of proinflammatory cytokines, including TNF-α, IFN-γ, IL-6, IL-8, IL-12, and IL-17 [20,21,22]. One strategy to reduce oxidative stress is to improve the antioxidant capacity and removal of peroxide products. Previous reports demonstrated that the regulation of cytokines as potential targets as well as the suppression of inflammatory responses and oxidative stress is associated with a pivotal part of the therapeutic strategies for treating UC [23,24]. This study indicated that FGR treatment reduced the levels of lipid peroxidation and increased the activity of the antioxidant defense system. Our study showed that FGR treatment also reduced the levels of TNF-α, IL-6, and IL-1B in serum and the expression of their respective mRNAs. Moreover, FGR treatment inhibited oxidative stress and macrophage infiltration by decreasing MPO activity in the DSS-induced colitis model. Therefore, these data demonstrated that FGR treatment alleviates DSS-induced colitis through balancing the secretion of inflammatory factors and regulating the intestinal permeability associated with an anti-inflammatory and antioxidant effect.

Research has shown that the cause of disease of UC is associated with a repetitive cycle of intestinal epithelial barrier disruption and inflammation. Accumulated intestinal epithelial cell apoptosis and dysfunction of tight junction proteins damages the physical intestinal barrier function [25,26] allowing increased intestinal permeability. Consequently, colonic inflammation can be induced by pathogenic antigens and bacterial invasion as well as by degradation of the tight junction through the triggering of a vicious cycle of release of pro- and anti-inflammatory factors [27,28]. Herein, we confirmed the epithelial cell apoptosis induction and disruption of tight junction proteins in DSS-exposed mice. Administration of FGR significantly inhibited colonic cell death. Additionally, FGR improved the intestinal barrier function by increased ZO-1 and occludin expressions as well as a claudin-2 decrease during epithelial damage. We found that FGR repaired the expression of tight junctions and their associated proteins as well as protected apoptosis in the treatment and prevention of colitis diseases.

Several reports have shown that the number of lymphocytes, macrophages, and neutrophils is increased in the DSS-induced acute colitis model [29,30]. Macrophages have an essential role in the production of inflammatory cytokines and mediators because of the secretion of TNF-α, IL-6, and IL-1β in immune responses [31]. Our study confirmed that the infiltration of macrophages, as shown by F4/80 staining, increased in DSS-treated mice, whereas this was inhibited by FGR treatment. Collectively, our results demonstrated that FGR alleviated inflammation by preventing the infiltration of macrophages into the intestinal crypts in DSS-induced colitis mice. Thus, this study proved that FGR can exert beneficial effects by improving intestinal barrier function and inhibiting inflammation and apoptosis in DSS-induced colitis mice.

IBD is a cluster of chronic remission inflammatory diseases that are associated with a variety of factors, including the gut microbiota, environment, and host genetics. DSS-induced colitis is frequently used as an animal model to investigate the pathological mechanisms of IBD. DSS causes damage to the IEC, allowing direct contact between the gut microbiota and host cells leading to intestinal inflammation. Symbiotic microbes are a key part of the gut barrier system, and an imbalance of microbes often increases the pathological response in the gut [32,33]. In this study, we investigated the protective effect of FGR extract for colonic homeostasis and preventing damage to the colon. Administration of FGR in mice resulted in dramatic changes in the gut microbiome, protected colonic epithelial cells and intestinal barrier against damaged by DSS, and attenuated DSS-induced colitis. Changes in colonic and barrier function were observed in the FGR-treated mice, and enhanced susceptibility to colonic inflammation was observed in control mice induced with DSS through feces, demonstrating that the regulatory role of FGR is highly dependent on the gut microbiota. Thus, our study suggests that FGR plays a protective role in DSS-induced colitis by maintaining the gut microbiome ecosystem providing new evidence for host–microbial interactions for FGR.

## 5. Conclusions

This study demonstrated that FGR extract relieves colitis by reducing the production of inflammatory mediators and increasing the activity of the antioxidant system to suppress tissue damage to the colonic mucosa in the DSS-induced colitis model. FGR could, therefore, be investigated for use in functional foods for the treatment of UC in humans. Finally, further research is needed to explore the interaction between FGR and target molecules to cure IBD; however, successful functional food development remains open-ended.

## Figures and Tables

**Table 1:** Bacterial 16s rRNA gene primers.

Bacteria		Primer Sequence	Annealing Temperature (°C)	Gene Information
Beneficial bacteria	*Lactobacillus acidophilus*	Fwd: AGCAGTAGGGAATCTTCCA	52	NCBI Genome UID: 1099
		Rev: CACCGCTACACATGGAG		
	*Bifidobacterium breve*	Fwd: CTCCTGGAAACGGGTGG	52	NCBI Genome UID: 1273
		Rev: GGTGTTCTTCCCGATATCTACA		
Harmful bacteria	*E. coli*	Fwd: GTTAATACCTTTGCTCATTGA	52	NCBI Genome UID: 167
		Rev: ACCAGGGTATCTAATCCTGTT		
	*Clostridium perfringens*	Fwd: GGGGGTTTCAACACCTCC	48	NCBI Genome UID: 158
		Rev: GCAAGGGATGTCAAGTGT		

## Data Availability

The data presented in this study are available upon request from the corresponding author.
